# Depression during the COVID-19 pandemic. A retrospective study on depressive disorders among psychiatric patients admitted at “elisabeta doamna” hospital galati, romania

**DOI:** 10.1192/j.eurpsy.2021.916

**Published:** 2021-08-13

**Authors:** O. Mihailov, I.D. Rădulescu, C. Moraru, P. Nechita, A.B. Ciubară, A. Ciubară

**Affiliations:** 1 Pediatric Pneumology, Hospital of Pneumophysiology “Saint Spiridon”Galati, Galati, Romania; 2 Psychiatrist, “Elisabeta Doamna” Psychiatric Hospital, Galati, Romania; 3 Resident Psychiatrist, Insitute of Psychiatry “Socola”, Iasi, Romania; 4 Corresponding Author, Md, Ph.d., Senior Psychiatrist, Insitute of Psychiatry “Socola”, Iasi, Romania; 5 Md, Ph.d, Associate Professor, Faculty of Medicine and Pharmacy, University “Dunarea de Jos”, Galati, Romania; 6 Md, Ph.d., Hab. Professor, Faculty of Medicine and Pharmacy, University “Dunarea de Jos” Head of Psychiatry Department, Senior Psychiatrist at ”Elisabeta Doamna” Hospital, Galati, Romania

**Keywords:** Covid-19, Depressive disorders, Pandemic

## Abstract

**Introduction:**

Isolation, life changes and increased stress lead to widespread concerns about the effects of the Covid-19 pandemic on psychiatric patients. The rise in depressive disorders is one of the negative effects associated directly and indirectly to the pandemic.

**Objectives:**

The purpose of this study was to investigate the impact of the COVID-19 pandemic on the prevalence of depressive disorders among the patients admitted to our hospital. The state of pandemia was declared on the 11th of March but it had already become a main stream media subject in our country at the beginning of the month with real life changes for our citizens.

**Methods:**

A retrospective study was performed at the Psychiatric Hospital ‘Elisabeta Doamna’ Galati, using the exact same period, between 01.03 and 30.09, in 2019 and 2020. ICD-10 criteria were used and pacients with either F32.x,F33.x or F38.x as discharge diagnosis were included.

**Results:**

In total, 7638 cases were admitted during the period in 2019, of which 751 (9,83%) had depressive disorders. In comparison with 2020 where out of 4050 admitted patients, the number had risen to 1034 (25,53%) a net increase in total number of cases by 37.6%.

**Conclusions:**

Analysis of the data shows a 2.5 times increase in the percentage of depressive disorders among our patients. Even taking in account the lower admition rates, we have seen a clear shift in the psychiatric profile of the average pacient and this has to be taken into consideration in the long and short term treatment of any psychiatric patient.

